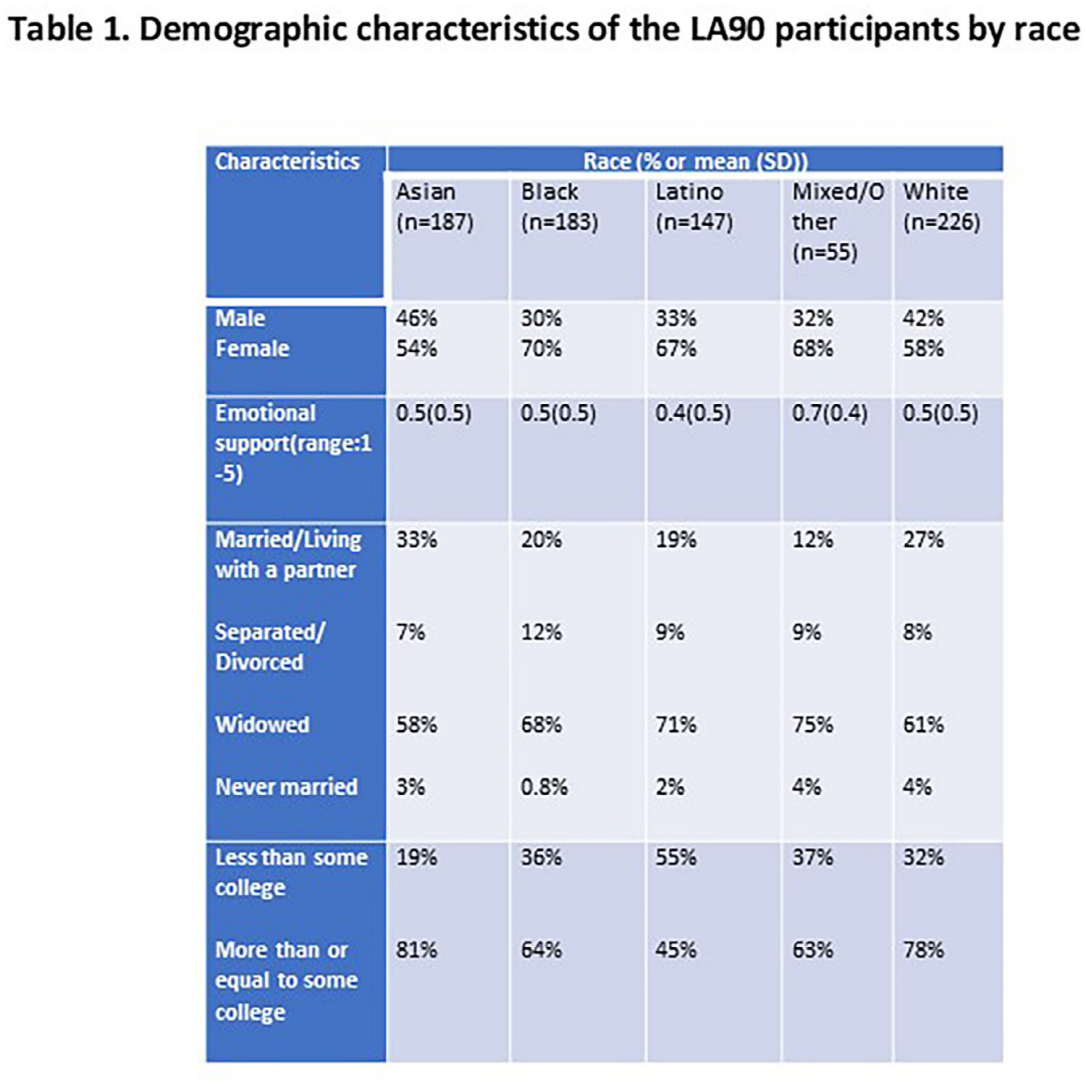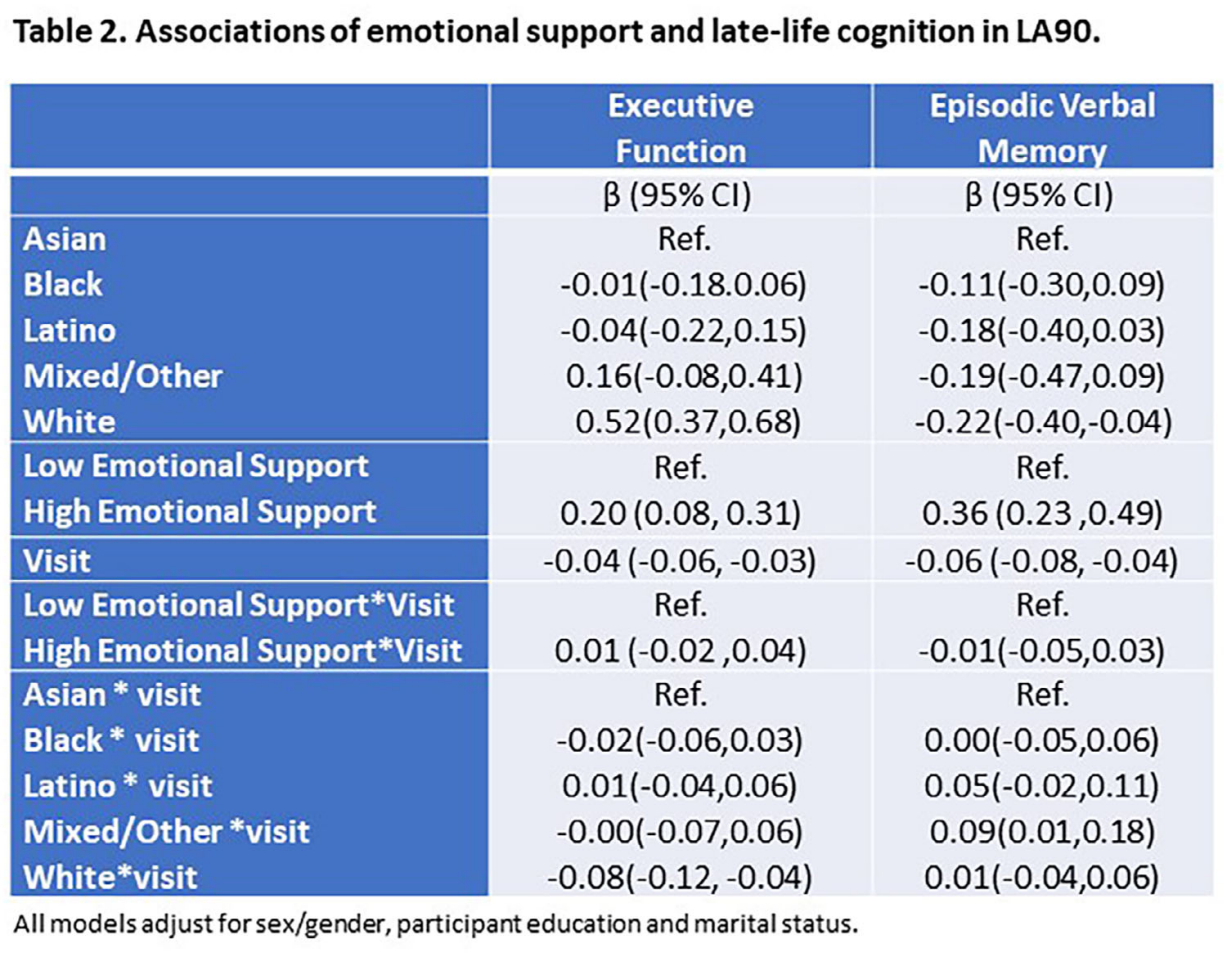# Racial/ethnic identity, emotional support and late life cognition and cognitive decline after age 90

**DOI:** 10.1002/alz70860_103626

**Published:** 2025-12-23

**Authors:** Kazi Sabrina Haq, María M. M. Corrada, Paola Gilsanz, Ruijia Chen, Rachel A. Whitmer, Rachel Peterson

**Affiliations:** ^1^ University of Montana, Missoula, MT, USA; ^2^ University of California, Irvine, Irvine, CA, USA; ^3^ Kaiser Permanente Northern California Division of Research, Pleasanton, CA, USA; ^4^ Boston University, Boston, MA, USA; ^5^ University of California, Davis, Davis, CA, USA

## Abstract

**Background:**

Disparities in late‐life cognition have been observed among diverse racial/ethnic groups, but the protective role of emotional support remains understudied among those at least 90 years old. Emotional support may help reduce racial disparities in cognitive function by fostering resilience. We examined if emotional support moderates the association between racial/ethnic identity and late‐life cognition and cognitive decline.

**Methods:**

*LifeAfter90* cohort (*n* = 814) participants responded to 16 questions assessing current emotional support using a 5‐option Likert scale. Participant scores (range: 1‐5) were classified as high (equal or greater than 4) vs. low (lower than 4) levels of emotional support. Cognition was assessed with the Spanish and English Neuropsychological Assessment Scales in 6‐month intervals (occurrences range 1‐7). Linear mixed effects models with random intercepts and slopes estimated associations between race/ethnicity, emotional support, and domain‐specific cognition (executive function and verbal episodic memory) at baseline and over time. Models were adjusted for sex, education and marital status.

**Results:**

Mean emotional support was highest for individuals who identified as mixed race or other (Mean = 0.7, SD=0.4) and lowest for Latinos (Mean = 0.4, SD=0.5; Table 1). In linear mixed effects models, higher emotional support was positively associated with baseline executive function (β=0.20 [95% CI: 0.08, 0.31]) and verbal episodic memory scores (β=0.36 [95% CI: 0.23, 0.49]; Table 2). There was no evidence of associations with cognitive decline (executive function β=0.01 [95% CI: ‐0.06, 0.04]; verbal episodic memory β=‐0.01 [95% CI: ‐0.05, 0.03]). Interactions between racial/ethnic identity and emotional support were non‐significant in models examining executive function (baseline interaction *p*‐value=0.27.; longitudinal interaction *p*‐value=0.72) and verbal episodic memory (baseline interaction *p*‐value=0.89; longitudinal interaction *p*‐value=0.32).

**Conclusions:**

Higher emotional social support is associated with better baseline executive function and verbal episodic memory in adults over age 90, but this effect does not vary by race/ethnicity. This finding suggests that public health interventions that facilitate emotional support may universally benefit the oldest‐old from diverse racial/ethnic backgrounds.